# Further evidence of the cross-reactivity of the Binax NOW® Filariasis ICT cards to non-*Wuchereria bancrofti* filariae: experimental studies with *Loa loa* and *Onchocerca ochengi*

**DOI:** 10.1186/s13071-016-1556-8

**Published:** 2016-05-05

**Authors:** Samuel Wanji, Nathalie Amvongo-Adjia, Abdel Jelil Njouendou, Jonas Arnaud Kengne-Ouafo, Winston Patrick Chounna Ndongmo, Fanny Fri Fombad, Benjamin Koudou, Peter A. Enyong, Moses Bockarie

**Affiliations:** Parasites and Vector Biology research unit (PAVBRU), Department of Microbiology and Parasitology, University of Buea, P.O. Box 63, Buea, Cameroon; Research Foundation for Tropical Diseases and the Environment (REFOTDE), P.O. Box 474 Buea, Cameroon; Centre for Neglected Tropical Diseases (incorporating the Lymphatic Filariasis Support Centre), Liverpool School of Tropical Medicine, Liverpool, UK

**Keywords:** ICT, Cross-reactivity, Non-*Wuchereria* proteins, In vitro and in vivo experimental models of filariae

## Abstract

**Background:**

The immunochromatographic test (ICT) for lymphatic filariasis is a serological test designed for unequivocal detection of circulating *Wuchereria bancrofti* antigen. It was validated and promoted by WHO as the primary diagnostic tool for mapping and impact monitoring for disease elimination following interventions. The initial tests for specificity and sensitivity were based on samples collected in areas free of loiasis and the results suggested a near 100 % specificity for *W. bancrofti*. The possibility of cross-reactivity with non-*Wuchereria bancrofti* antigens was not investigated until recently, when false positive results were observed in three independent studies carried out in Central Africa. Associations were demonstrated between ICT positivity and *Loa loa* microfilaraemia, but it was not clearly established if these false positive results were due to *L. loa* or can be extended to other filarial nematodes. This study brought further evidences of the cross-reactivity of ICT card with *L. loa* and *Onchocerca ochengi* (related to *O. volvulus* parasite) using in vivo and in vitro systems.

**Methods:**

Two filarial/host experimental systems (*L. loa*-baboon and *O. ochengi-*cattle) and the in vitro maintenance of different stages (microfilariae, infective larvae and adult worm) of the two filariae were used in three experiments per filarial species. First, whole blood and sera samples were prepared from venous blood of patent baboons and cattle, and applied on ICT cards to detect circulating filarial antigens. Secondly, larval stages of *L. loa* and *O. ochengi* as well as *O. ochengi* adult males were maintained in vitro. Culture supernatants were collected and applied on ICT cards after 6, 12 and 24 h of in vitro maintenance. Finally, total worm extracts (TWE) were prepared using *L. loa* microfilariae (Mf) and *O. ochengi* microfilariae, infective larvae and adult male worms. TWE were also tested on ICT cards. For each experiment, control assays (whole blood and sera from uninfected babon/cattle, culture medium and extraction buffer) were performed.

**Results:**

Positive ICT results were obtained with whole blood and sera of *L. loa* microfilaremic baboons, culture supernatants of *L. loa* Mf and infective larvae as well as with *L. loa* Mf protein extracts. In contrast, negative ICT results were observed with whole blood and sera from the *O. ochengi-*cattle system. Surprisingly, culture supernatant of *O. ochengi* adult males and total worm extracts (Mf, infective larvae and adult worm) were positive to the test.

**Conclusions:**

This study has provided further evidence of *L. loa* cross-reactivity for the ICT card. All stages of *L. loa* seem capable of inducing the cross-reactivity. *Onchocerca ochengi.* can also induce cross-reactivity in vitro, but this is less likely in vivo due to the location of parasite. The availability of the parasite proteins in the blood stream determines the magnitude of the cross-reactivity. The cross-reactivity of the ICT card to these non-*W. bancrofti* filariae poses some doubts to the reliability and validity of the current map of LF of Central Africa that was generated using this diagnostic tool.

**Electronic supplementary material:**

The online version of this article (doi:10.1186/s13071-016-1556-8) contains supplementary material, which is available to authorized users.

## Background

Lymphatic filariasis (LF) is a parasitic disease caused by nematodes (*Wuchereria bancrofti, Brugia malayi* and *Brugia timori*) whose preferred habitats are the lymphatic vessels and lymph nodes where they induce the development of disfiguring and debilitating clinical symptoms in some individuals [1]. The infection is transmitted by various genera of mosquitoes [2]. LF is considered to be one of the health problems of greatest social and economic impact in endemic areas [3]. While no human-infecting species of *Brugia* occur in Africa, *W. bancrofti* is responsible of more than 45 million LF cases in the sub-Saharan Africa, and this parasite coexists with four other filarial parasites infecting humans in central Africa: *Loa loa*, *Onchocerca volvulus*, *Mansonella perstans* and *M. streptocerca* [4].

Circulating microfilariae (Mf) are responsible for the transmission, therefore the most practical and feasible method of interrupting transmission is rapid reduction of microfilarial load in the communities by annual mass drug administration (MDA) of antifilarial drugs such as diethylcarbamazine (DEC) and albendazole. It is based on this principle that the Global Alliance for the Elimination Lymphatic Filariasis (GAELF) was developed [5–7]. However, the co-endemicity of *W. bancrofti* and *L. loa* in central Africa has been a major preoccupation for programmes to eliminate LF. This programme is based on the determination of the prevalence of infection using rapid assessment procedures [8–10] and mass administration of preventive chemotherapy with a combination of ivermectin and albendazole for bancroftian filariasis in areas where *L. loa* is absent or exists at a low level, or biannual treatment regimen of albendazole in areas where *L. loa* is highly endemic.

Diagnosis of bancroftian filariasis relied until recently almost exclusively on the microscopic identification of Mf in night blood films [11–14]. Apart from the low sensitivity of this method, it is also inconvenient in terms of the time of blood collection, since Mf are more present in the peripheral blood at night (22:00–02:00 h) [15, 16]. Tests based on the detection of *W. bancrofti* circulating filarial antigen (CFA) have been widely used for LF mapping and are commercially available in kit formats [17–20]. The great advantage of using CFA for diagnosis is that CFA levels remain stable during the 24 h of the day, avoiding the need to collect blood samples at night. A friendly use immunochromatographic card test produced by BINAX (known as NOW® Filariasis ICT kit) detects CFA in serum and whole blood specimens [18, 21]. Despite the fact that the monoclonal antibody (mAb AD12) used in this test has been generated from *Dirofilaria iminitis* antigen, the ICT was demonstrated to be sensitive and specific to adult *W. bancrofti* CFA [18]. ICT has been the main tool for the rapid assessment of bancroftosis worldwide [22–25].

Reports from the Democratic Republic of Congo and Cameroon have recently demonstrated association between the ICT test results and *L. loa* microfilaraemia in individuals negative for *W. bancrofti* Mf [26–28]. In the Democratic Republic of Congo, CFA positivity was strongly associated with high *L. loa* microfilarial counts detected in the night blood smears. The Cameroon studies confirmed the strong association between the ICT positivity and *L. loa* intensity (Mf/ml blood) at the individual level using day blood smears. Furthermore, data from Cameroon demonstrated that ICT positivity is strongly associated with high *L. loa* prevalence. These results suggest that the main confounding factor for positive ICT test card results are the high levels of *L. loa*.

Despite this high incrimination of *L. loa* as the major confounder in ICT cross-reactivity, several questions, with potential epidemiological implications for the development of the new mapping tools to be used in potential areas of co-endemicity LF/other filariases, need answers. It would be good to know more about the proteins that are responsible for cross-reactivity. Are they present in different stages of *L. loa*? Are they somatic proteins, or excreted/secreted proteins? Are they also present in *Onchocerca* spp.? We designed this study with the overall objective to answer the above questions and provide further evidence of the cross-reactivity of the Binax NOW® Filariasis ICT cards to non-*Wuchereria bancrofti* antigens using *Loa loa* and *Onchocerca ochengi* as experimental models*.*

## Methods

### Experimental design

Two filarial-host systems (baboon-*L. loa* and cattle-*O. ochengi*) and the in vitro maintenance of the different stages of the two filariae were used in the six experiments described below.

#### Experiment 1: Baboon - *L. loa* system (Fig. 1)

**Fig. 1:**
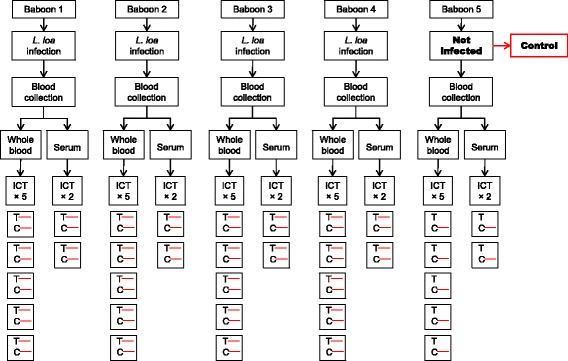
Experimental design of the filariasis test using whole blood and serum from *L. loa* microfilaremic baboons. Visible C and T lines indicate a positive result; visible C line only refers to a negative result

This experiment involved collecting venous blood from patent Baboons and non-infected Baboon. The infection and follow up of these animals were previously described [29]. Whole blood and sera from each animal were applied to the ICT card. Whole blood ICT was performed with four *Loa*-microfilaremic baboons and one control animal (not infected). For each animal, the test was repeated five times. Simultaneously, sera samples were prepared from each baboon and duplicate tests were done for the detection of CFA.

#### Experiment 2: In vitro culture of *L. loa* microfilariae and infective larvae and application of supernatant of culture to ICT card test (Fig. 2a)

**Fig. 2:**
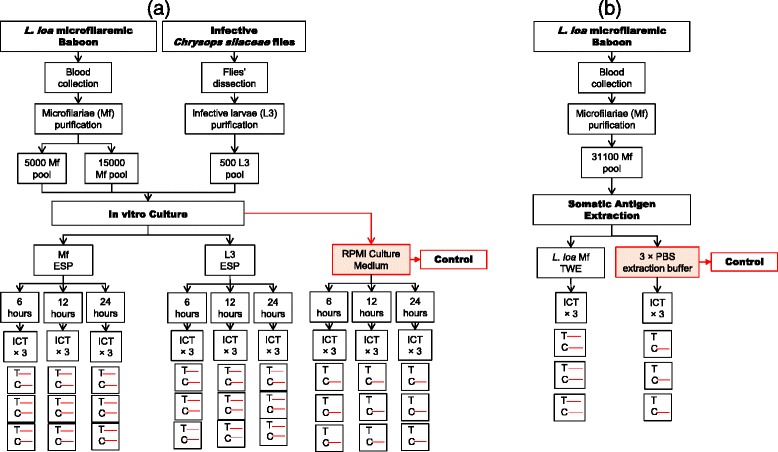
Immunochromatographic test using **a** culture supernatant and **b** somatic antigens of different life-cycle stages of *L. loa*. Visible C and T lines indicate a positive result; visible C line only refers to a negative result. *Abbreviations*: ESP, excretory-secretory product; TWE, total worm extract

*Loa loa* Mf and infective larvae (L3) were isolated and purified from patent baboon venous blood and infected *Chrysops*, respectively. Pools of parasites (5,000 Mf, 15,000 Mf and 500 L3) were cultured in vitro. The culture supernatant (ESP) was used on three ICT cards test at three time points of culture (6, 12 and 24 h).

#### Experiment 3: extraction of somatic protein from *L. loa* microfilariae and application to ICT card test (Fig. 2b)

Total proteins were extracted from a batch of 31,100 *L. loa* Mf. The total worm extracts (TWE) were tested on ICT card, in triplicate.

#### Experiment 4: Cattle - *O. ochengi* system

**Fig. 3:**
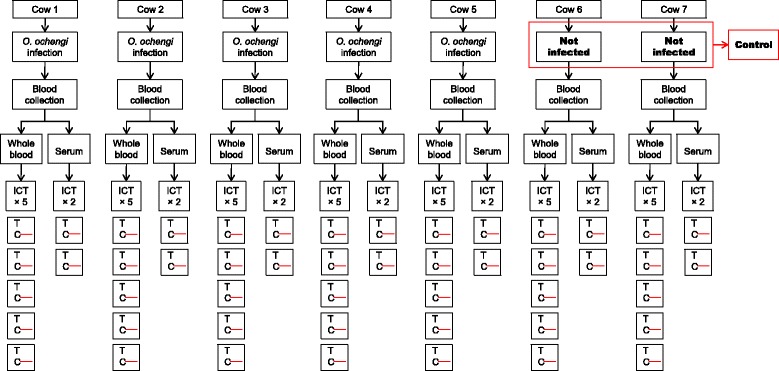
Experimental design of the filariasis test using whole blood and serum from *O. ochengi* microfilaridermic cattle. Visible C and T lines indicate a positive result; visible C line only refers to a negative result

The experiment involved collecting venous blood from cattle naturally infected with *O. ochengi* and from cattle born in area of non-transmission of *O. ochengi*. Both whole blood and sera prepared from venous blood were applied to the ICT card test (Fig. 3). Whole blood experiments were repeated five times while sera were applied in duplicates.

#### Experiment 5: In vitro culture of *O. ochengi* microfilariae, infective larvae and adult male worms and application of culture supernatant to the ICT test card (Fig. 4a)

**Fig. 4:**
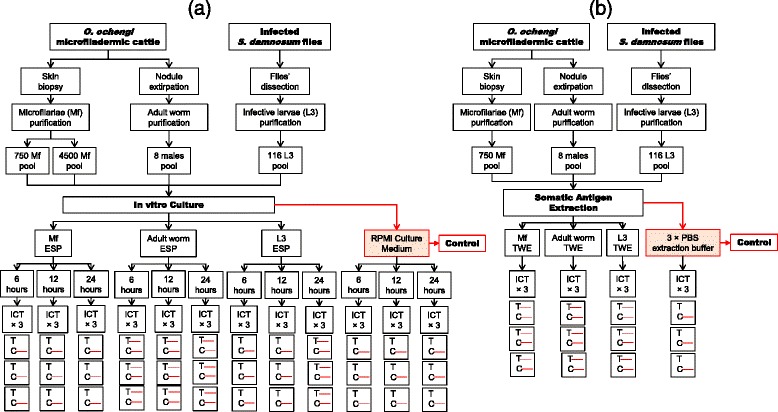
Immunochromatographic test using **a** culture supernatant and **b** somatic antigens of different life-cycle stages of *O. ochengi*. Visible C and T lines indicate a positive result; visible C line only refers to a negative result. *Abbreviations*: ESP, excretory-secretory product; TWE, total worm extract

Mf, adult worms and L3 were isolated from skin biopsy, nodules and *Simulium damnosum* vectors, respectively. Each parasite stage was pooled (750 Mf, 4,500 Mf, 8 adult males and 116 L3) and maintained in in vitro culture. The culture supernatant (ESP) was used on three ICT cards test at three time points of culture (6, 12 and 24 h).

#### Experiment 6: Extraction of somatic protein from *O. ochengi* microfilariae, adult male and infective larvae and application to ICT test card (Fig. 4b)

Total protein extracts were prepared from 50,000 Mf, 84 adult males and 230 L3. The total worm extracts (TWE) were tested on ICT card in triplicate.

### Ethical considerations

Ethical and administrative clearances for the use of baboons and cattle in this study were obtained from the Ministry of Scientific Research and Innovation of Cameroon (Research permit #028/MINRESI/B00/C00/C10/C12). The animal procedures were conducted in accordance with the guidelines with animal care and use committee at the National Institutes of Health (USA) and University of Georgia, Athens, USA. The use of non-human primates for research was approved by the Committee on the Ethical Use of Animals in Research (CEUAR) within the Research Foundation for Tropical Diseases and Environment (REFOTDE), Cameroon. All relevant aspects of the International Primatological Society (IPS) 2007 guidelines on the acquisition, care and breeding of non-human primates for research were followed [29, 30].

### Detection of CFA in whole blood and sera samples using Binax NOW® Filariasis ICT

From each group of animals, 5 ml of venous blood were aseptically collected between 8 and 11 am. ICT was performed using 100 μl of heparinized capillary blood according to the manufacturer’s instructions. Whole blood ICT were repeated 5 times to assess the reproducibility of the test. The remaining blood (approximately 4 ml) was equally distributed into two vacuum tubes containing EDTA and used for the sera preparation and parasite (*L. loa* Mf only) purification, respectively. A blood vacuum was centrifuged at 1,150 × *g* at 20 °C with a bench centrifuge Humax 14 K (Human, Germany), and sera were separated. A 100 μl of each serum was placed on the ICT sample pad and the test was performed in duplicate as previously described. All readings were made within the time limits (10 min after the migration of the blood samples) and the results pictured and recorded.

### Culture medium

The incomplete culture medium (ICM) used for parasite culture was made up of Roswell Park Memorial Institute (RPMI 1640) supplemented with 2 g/l sodium bicarbonate, 10 μg/ml Fluconazole (Sigma-Aldrich, Sintra, Portugal), 100 U/ml penicillin, 100 μg/ml streptomycin, and 200 μg/ml neomycin (Gibco, Life Technologies, Grand Island, NY, USA).

### Parasite material

#### Isolation of *L. loa* Mf from blood samples

Microfilariae were extracted using iso-osmotic Percoll extraction method previously described by Van Hoegaerden & Ivanoff [31] with some modifications. Briefly, an aliquot of 2 ml of undiluted blood was pipetted and layered gently onto a Percoll gradient (40, 50 and 65 %) in a 15 ml centrifuge tube and spin at 4,000 × *g* for 10 min at 25 °C. The layer of Percoll gradient containing microfilariae (located above the buffy coat) was removed using a syringe and filtered gently through a 5 μm pore-size cellulose filter. The filter was then transferred immediately to a Petri dish containing culture medium and incubated at 37 °C for 5 min. Thereafter, the microfilariae were concentrated by centrifugation (350 *g* for 10 min at 25 °C) and quantified using an inverted microscope (Motic AE21).

#### Isolation of *L. loa* and *O. ochengi* infective larvae (L3) from flies

##### *Loa loa infective larvae*

Engorged *Chrysops* spp. flies were trapped from loiasis endemic areas and transported to the laboratory where they were maintained for 14 days (time for the microfilariae to develop to L3 stage). During this time, flies were fed with 15 % sucrose at 23 °C. A total of 1,600 were dissected in several batches and used. The flies were drowned in sterile drowning solution (sterile distilled water + 0.2 % Tween 20) in a sterile Petri dish for 1 min, transferred to a cleaning solution (sterile distilled water) and placed into a Petri dish containing dissecting medium (ICM) using a sterile forceps. The head and thorax were separated from the abdomen, placed in different Petri dishes and dissected with sterile needles under a dissecting microscope. Any emerging larvae were collected and transferred with a sterile Pasteur pipette into the first of 3 wells of a depression or concentration plate filled with sterile medium. At the end of the dissection, the larvae were washed 3 times by transferring them from one depression well to another, then from the third into a new centrifuge tube containing dissection medium with a sterile Pasteur pipette [32, 33]. They were then preserved in CCM supplemented with 10 % calf serum until the experiment.

##### *Onchocerca ochengi infective larvae*

*S. damnosum* flies that had fed to repletion on *O. ochengi*-microfilaridermic cattle were caught, kept individually in rearing tubes and transported to the laboratory. The flies were maintained for 10 days with 15 % sucrose at 23 °C with the mortality rate being recorded daily. The surviving flies were dissected according to the protocol described above with slight modifications [32, 33] and the recovered parasites were immediately placed into the culture.

#### Isolation of *O. ochengi* Mf and adult males from cattle umbilical skin

##### *Onchocerca ochengi* Mf

The umbilical skin of the cattle was removed and placed in a Petri dish containing ICM incubated for 4 h at 37 °C. After incubation, Mf migrated from the skin into the medium. The medium was filtered using a nylon cloth attached round to a half cut centrifuge tube into another Petri dish, then transferred into centrifuge tubes. Mf were washed in RPMI (ICM) twice by centrifugation for 10 min at 1,500 rpm.

##### *Onchocerca ochengi adult males*

Cattle skin collected from a local slaughter house was transported on ice to the laboratory within an hour. In the laboratory, the skin was properly washed with tap water, placed on a dissecting board, shaved with a surgical blade, rinsed with tap water, dabbed with a clean cloth to eliminate excess moisture and sprayed entirely with 70 % alcohol. The nodules were found by palpation and carefully removed from the skin with a surgical blade. The nodules were then gently opened and placed in individual Petri dishes containing ICM, and incubated at 37 °C for 4 h. Following incubation, any whole adult worm that migrated into the medium was placed in a new Petri dish containing ICM which was observed under a dissecting microscope to check the viability. *Onchocerca ochengi* adult males were washed 3 times by transfer into new Petri dishes containing sterile medium. Isolated parasites were quantified using an inverted microscope. An aliquot of each parasite was used for culture while the remaining were frozen for the preparation of total worm extract.

### In vitro culture

*Loa loa* (Mf and L3) and *O. ochengi* (Mf, L3 and male adult worm) filariae were cultured in 400 μl RPMI per well in 48-well flat bottom culture plates. The plates were incubated at 37 °C, 5 % CO_2_, for 48 h in a CO_2_ incubator (Shel Lab). Each sample was cultured in 4 wells and only one well per sample was used for CFA testing at a given time point post-culture (6, 12 and 24 h). An aliquot of 100 μl culture medium supernatant was carefully collected from each well at 6, 12 and 24 h post-culture and applied on ICT card. Experiments were performed in duplicates and the results were read according to the manufacturer’s instructions.

### Preparation of total worm extract (TWE)

Adult worms (84), Mf (50,000) and L3 (230) of *O. ochengi* and Mf (31,100) of *L. loa* were rinsed 3 times in PBS by centrifugation at 1,500 rpm for 10 min. They were placed in an ice cold ceramic mortar containing diethylaminoethyl beads (DEAE beads, GE Healthcare Life Sciences, London, UK) and ground using a pestle. A micro-volume of PBS (500–1,000 μl) was added progressively while grinding. The mixture was kept at 4 °C for 4 h, then transferred in micro-centrifuge tubes and centrifuged for 15 min at 13,500 rpm (4 °C) with a refrigerated micro-centrifuge (Eppendorf, Ocala, FL, USA,). The supernatants were collected into Eppendorf tubes and frozen at -20 °C until used within a week. The amount of proteins in the homogenate was assessed using quantitative micro-assay based on the method of Bradford [34]. The samples were then diluted to 5 μg/ml with PBS except for those of *L. loa* mf because their concentration was low Bradford [34]. The samples were then diluted to 5 μg/ml with PBS except for *Loa loa* Mf due to its low concentration (1 μg/ml). A 100 μl of TWE was pipetted onto the sample pad of the ICT, and duplicate testing was performed as previously described.

### Expression of results

Positive and negative results were based on visual observation of the test.

## Results

### Whole blood and sera samples ICT results

Overall, 70 and 24 card tests were used for the detection of CFA in whole blood and sera samples respectively (Figs. 1, 3). All *Loa*-microfilaremic baboons were found positive by ICT. Rather, *O. ochengi* microfilaridermic cattle were tested negative. In both animal group, controls (uninfected animals) had negative results. Tables 1 and 2 summarise animals' infection history and ICT results.

**Table 1:** Baboon infection history and whole blood/sera ICT results

Baboon SN	Date of first inoculation	Date of second inoculation	Number of L3 inoculated	Patency period (days)	Highest *Loa* mf load	Mf count on analysis	ICT results	
							Whole blood^b^	Serum^c^
1	14.iv.2006	21.xii.2011	600	184	17,900	40	Positive	Positive
2	21.x.2008	22.xii.011	600	174	150,000	2,480	Positive	Positive
3	23.xii.2011	None	600	206	190,380	42,220	Positive	Positive
4	27.ix.2012	None	600	Natural infection^a^	9,213	3,980	Positive	Positive
5 (Control)	None	None	None	0	0	0	Negative	Negative

^a^Animal with positive *L. loa* infection prior to its introduction in the rearing centre

^b^ICT done 5 times

^c^ICT done in duplicates

**Table 2:** Cattle infection history and whole blood/sera ICT results

Cow SN	Origin (*O. ochengi* endemicity)	Approximate number of nodules	Average Mf/skin snip	ICT results	
				Whole blood ^a^	Serum ^b^
1	Endemic	100	92	Negative	Negative
2	Endemic	30	7.5	Negative	Negative
3	Endemic	30	4,5	Negative	Negative
4	Endemic	10	35	Negative	Negative
5	Endemic	10	137	Negative	Negative
6 (control)	Non-endemic	0	0	Negative	Negative
7 (control)	Non-endemic	0	0	Negative	Negative

^a^ICT done 5 times

^b^ICT done in duplicates

### ICT results with in vitro culture supernatants

Various loads of *O. ochengi* and *L. loa* stages were cultured for 24 h and the results of culture supernatants tested with ICT cards at different time intervals are given in Table 3. The positive reactivity of the test differs depending on parasites species and stages, number of parasites cultured per well and the time of incubation (Additional file 1).

**Table 3:** ICT results of culture supernatants of different stages of *L. loa* and *O. ochengi*

Parasite	Stage	No. of parasites per well	Incubation time (h)	ICT result
*L. loa*	Mf	5,000	6	+
		150,00	6	++
	L3	500	6	++
*O. ochengi*	Mf	750	12	_
		4,500	6	_
		4,500	24	_
	L3	116	6	_
		116	24	+
	Adult male	8	6	++
		8	12	+++
Control RPMI			24	-

-, negative; +, positive

*Loa loa* Mf and L3 were very active (motile) under the same conditions, and the ICT test was positive for wells with 5,000 Mf cultured for 6 h. The test band was prominently coloured when the number of Mf cultured were15, 000 per well. The supernatant from *L. loa* infective larvae (500 L3/well) after 6 h incubation, was also positive with a strong band (Fig. 2a). In contrast, *O. ochengi* species, only wells with 8 adult males incubated for 6 h tested positive with thick bands, while wells with up to 4,500 Mf and 116 L3 incubated for 24 h tested negative (Fig. 4a). In addition, it was observed that less than 80 % of *O. ochengi* Mf or L3 survived for 24 h in RPMI without serum supplement. The parasites were either sluggish or immotile after 24 h incubation.

### ICT results with TWE

The total proteins were extracted from the experimental parasites and tested on ICT (Figs. 2b, 4b and Table 4). In contrast to what was observed with the culture supernatant, the extract from almost all parasite stages tested positive with ICT card (Additional file 2).

**Table 4:** ICT result of total worm extract of different life-cycle stages of *L. loa* and *O. ochengi*

Parasites	Protein concentration (μg/ml)	Life-cycle stages	ICT result
*L. loa*	1	Mf	+
*O. ochengi*	5	Mf	++
	5	L3	++
	5	Adult male	++
Control PBS			-

-, negative; +, positive

## Discussion

Previous studies demonstrated high incrimination of *L. loa* as the major confounder in ICT cross-reactivity [26–28] but none of them established causal relationship between *L. loa* and the cross-reactivity. In addition, several questions with potential epidemiological implications for the development of the new mapping tools to be used in potential areas of co-endemicity LF/other filarial species were pending answers. The present study was designed to provide further evidence of the cross-reactivity of the Binax NOW® Filariasis ICT Cards to non-*W. bancrofti* filariae proteins using experimental models (in vitro and in vivo) with *L. loa* and *O. ochengi* filariae.

One of the specific objectives of this study was to demonstrate that there is a causal relationship between *L. loa* and the ICT positivity. In epidemiology, several models of causal relationship exist between an exposure and an outcome. An exposure may be necessary and sufficient to induce the outcome; the exposure may be necessary but not sufficient, it could be sufficient but not necessary, or it could neither be sufficient or nor necessary to induce the outcome [35–37]. By using a system in which naïve baboons were experimentally infected with *L. loa* infective larvae and kept in captivity free from any other parasitic infection, we demonstrated that whole blood and sera from animals with *L. loa* infection alone can induce positivity to ICT card test. *Loa loa* therefore, is sufficient in inducing positivity to the ICT card. These findings have several practical and operational epidemiological implications. In an area non-endemic for *W. bancrofti* where *L. loa* is present, the use of ICT card test to assess the endemicity of lymphatic filariasis will produce false and misleading information on the existence of LF [27, 28]. The map generated in such situation would likely be very similar to the *L. loa* map. The recent map of bancroftian filariasis of Cameroon generated with ICT test card is one example illustrating one such scenario [38]. In an area co-endemic for *L. loa* and *W. bancrofti*, the map generated using ICT card will still be misleading as the level of infection due to *W. bancrofti* will be inflated with negative effects on the overestimation of the number of persons to be treated and the duration of the treatment. The only situation when the bancroftian filariasis map generated by ICT card test is likely to be truthful is in areas where *L. loa* is absent [23, 24, 39, 40].

Using in vitro culture system of microfilariae and infective larvae, the study has demonstrated that products from infective larvae and Mf of *L. loa* could induce positivity to the ICT. Even though we did not culture the adult stage of *L. loa*, it is possible that this stage would also produce cross-reactive antigen and thus needs further investigation. Total proteins (somatic) extracts from each of the two stages of *L. loa* also induced ICT positivity. This gives room for speculation about the mode of production and stockade of the proteins responsible for cross-reactivity. These proteins may be first produced and stocked in vesicles and released progressively by the worms into the blood stream or the lymphatic system. Although this reasoning is speculative for now, it has important implications for the capacity of a filarial species to induce cross-reactivity [41, 42]. The adult worms or microfilariae should be located with direct contact to the blood system or the lymphatic system to produce sufficient material that can be detected in the blood [4]. This implies that a filarial species whose location is not in direct contact with the blood or lymphatic system may produce proteins that might not be in sufficient quantity in the blood to induce cross-reactivity. Indeed, both microfilariae and adult stages of *L. loa* benefit from locations (aponephrotic of the connective tissue between muscles layers for adults and blood streams for microfilariae) that are compatible with our hypothesis. In further support to this argument, whole blood and sera from cattle with patent infection with *O. ochengi* and reasonable number of palpable nodules did not show any positivity to the ICT card. Meanwhile, the supernatant of adult *O. ochengi* maintained in culture gave high positivity to the test. *Onchocerca ochengi* are located in subcutaneous nodules and their ES products may not naturally be in contact with the blood stream. It might therefore be difficult to have enough concentration of antigenic proteins from nodule that can induce positivity to ICT by the whole blood and sera samples of *O. ochengi* infected cattle. In this study, we were not able to induce ICT positivity, even with incubation of 4,500 Mf of *O. ochengi* for 24 h. This contrasts with *L. loa* Mf that, within similar experimental conditions, generate positivity to the test. These findings indicate differences in the capacity of Mf from several filarial species to produce proteins responsible of cross-reaction with the ICT. These data from experimental studies can help the interpretation of some epidemiological observations. Indeed, despite the intensive use of ICT test in areas of co-endemicity *W. bancrofti*/*O. volvulus* in West and East Africa, there have never been a report of cross-reactivity of the test to *O. volvulus* [40, 43].

## Conclusion

This study has provided further evidence of *L. loa* cross-reactivity to the ICT card. All stages of *L. loa* seem capable of inducing the cross-reactivity. *Onchocerca* spp. can also induce cross-reactivity in vitro, but this is less likely in vivo due to the unfavourable location of parasites and their secreted products. The availability of the parasite proteins in the blood stream determines the magnitude of the cross-reaction. The cross-reactivity of the ICT card to these non-*W. bancrofti* filariae reinforces doubts on the validity of the current map of LF in central Africa that was generated using this diagnostic tool and call for development and validation of new tools for the mapping of LF in this region.

## Additional files

Additional file 1: Figure S1. Photograph of ICT cards after testing the ES product of *L. loa* and *O. ochengi* Mf, L3 and adult. (JPG 85 kb) Additional file 2: Figure S2. Photograph of ICT cards after testing the TWE of *L. loa* and *O ochengi* Mf, L3 and adult. (JPG 52 kb)

## Abbreviations

CFA: circulating filarial antigens
ES: excretory-secretory
GAELF: global alliance to eliminate lymphatic filariasis
ICT: immunochromatographic card test
L3: Third stage/infective larvae
LF: lymphatic filariasis
MDA: Mass drug administration
Mf: microfilariae
TWE: total worm extract
