# Supplementary figures and images for: Further evidence of the cross-reactivity of the Binax NOW® Filariasis ICT cards to non-Wuchereria bancrofti filariae: experimental studies with Loa loa and Onchocerca ochengi
Source: Parasit Vectors. 2016 May 5;9:267. doi: 10.1186/s13071-016-1556-8 (PMC4858834; doi:10.1186/s13071-016-1556-8)

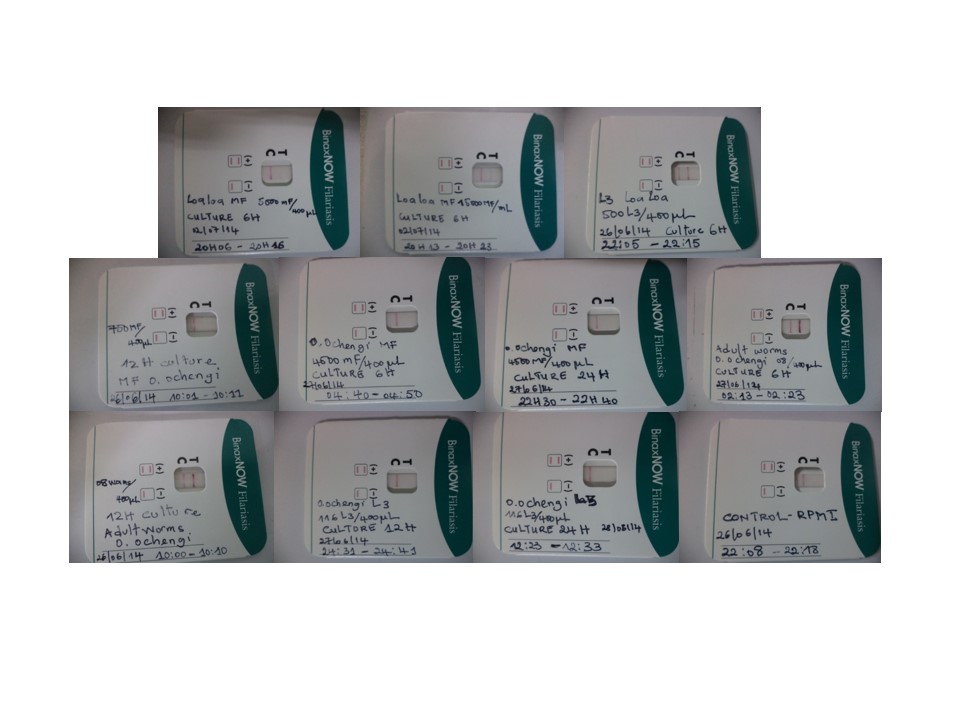

Supplement: Additional file 1: Figure S1. — Photograph of ICT cards after testing the ES product of L. loa and O. ochengi Mf, L3 and adult. (JPG 85 kb) [file 13071_2016_1556_MOESM1_ESM.jpg]

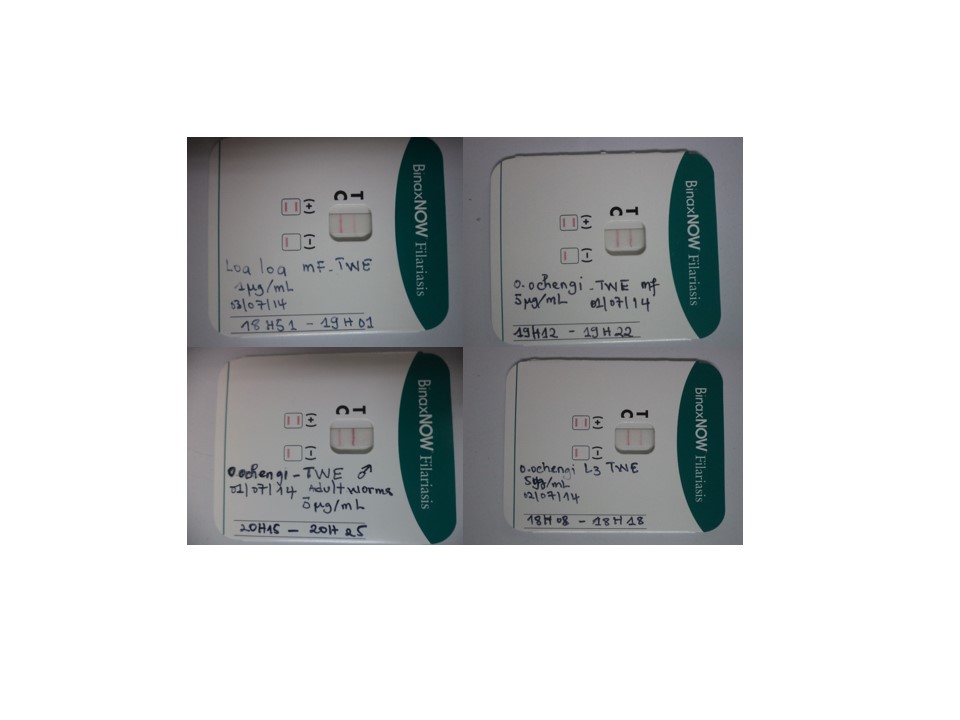

Supplement: Additional file 2: Figure S2. — Photograph of ICT cards after testing the TWE of L. loa and O ochengi Mf, L3 and adult. (JPG 52 kb) [file 13071_2016_1556_MOESM2_ESM.jpg]
